# Mosquitoes as Vectors of *Mycobacterium ulcerans* Based on Analysis of Notifications of Alphavirus Infection and Buruli Ulcer, Victoria, Australia

**DOI:** 10.3201/eid3009.231073

**Published:** 2024-09

**Authors:** Andrew H. Buultjens, Ee Laine Tay, Aidan Yuen, N. Deborah Friedman, Timothy P. Stinear, Paul D.R. Johnson

**Affiliations:** Doherty Institute, University of Melbourne, Melbourne, Victoria, Australia (A.H. Buultjens, T.P. Stinear);; Victoria Department of Health, Melbourne (E.L. Tay, A. Yuen, N.D. Friedman);; Austin Health, Heidelberg, Victoria, Australia (P.D.R. Johnson);; University of Melbourne, Melbourne (P.D.R. Johnson)

**Keywords:** *Mycobacterium ulcerans*, tuberculosis and other mycobacteria, Buruli ulcer, vector-borne infections, mosquito-borne diseases, viruses, alphavirus infections, Australia

## Abstract

Alphavirus infections are transmitted by mosquitoes, but the mode of transmission for *Mycobacterium ulcerans*, which causes Buruli ulcer, is contested. Using notification data for Victoria, Australia, during 2017–2022, adjusted for incubation period, we show close alignment between alphavirus and Buruli ulcer seasons, supporting the hypothesis of mosquito transmission of *M. ulcerans*.

Victoria, Australia, has been experiencing an intensifying outbreak of Buruli ulcer (*1*). The mode of transmission for *Mycobacterium ulcerans*, which causes Buruli ulcer, has been contested. The first evidence that mosquitoes might transmit the causative organism, *Mycobacterium ulcerans*, to humans was published in 2007 (*2*,*3*). Contemporaneous research also reported that native possums were a key environmental reservoir, shed *M. ulcerans* in their excreta, and frequently suffer from Buruli ulcer themselves. Combining those facts, we proposed that mosquitoes transmit *M. ulcerans* from possums to humans and likely between possums (*4*). 

Further reports from Victoria published in April 2009 (*5*) and December 2021 (*6*) considered correlations between patterns of annual notified cases of Ross River and Barmah Forest viruses (alphavirus infections) and Buruli ulcer. A positive correlation would be expected if Buruli ulcer were mosquito transmitted, as has been established for alphavirus infections. The 2009 report (*5*) identified a partial correlation between annual notifications of alphavirus infections and Buruli ulcer during 2002–2008, consistent with the mosquitoborne transmission hypothesis. However, the 2021 report (*6*) indicated no ongoing statistical association in annual notifications since 2008 using linear statistical methods and concluded that factors other than mosquitoes were likely behind the change in Buruli ulcer incidence in Victoria. We hypothesized that a new analysis of notification data during a period of higher Buruli ulcer incidence using a nonlinear statistical approach applied this time to monthly instead of annual notifications would help to resolve the question of transmission vectors so that we could focus and intensify our Buruli ulcer prevention efforts at a public health level. 

Separate ethics approval was not required because notification data in this study were collected and used under the legislative authority of the Public and Wellbeing Act of 2008, and we used only aggregated, deidentified data. Data were summated by month and accessed with permission and assistance from senior epidemiologists at the Victoria Department of Health. 

## The Study

Victoria, the smallest of Australia’s mainland states, has a temperate southern hemisphere climate with distinct seasons. Officially, summer in Victoria is defined as December–February, autumn as March–May, winter as July–August, and spring as September–November. 

Buruli ulcer was initially made legally notifiable to the Department of Health in Victoria in January 2004, but notifications have markedly increased only in more recent years. The surveillance definition for Buruli ulcer in Victoria has been updated recently (*7*). Cases of alphavirus infection, classified according to national surveillance definitions, have been nationally notifiable for many years. In collaboration with colleagues at the Victoria Department of Health, we analyzed all notification data from Victoria for cases of both Buruli ulcer and alphavirus infection (Ross River virus and Barmah Forest virus infections combined) by month for the 6 calendar years 2017–2022 (Appendix). We assumed month of notification to be the same as month of diagnosis. 

**Figure 1:**
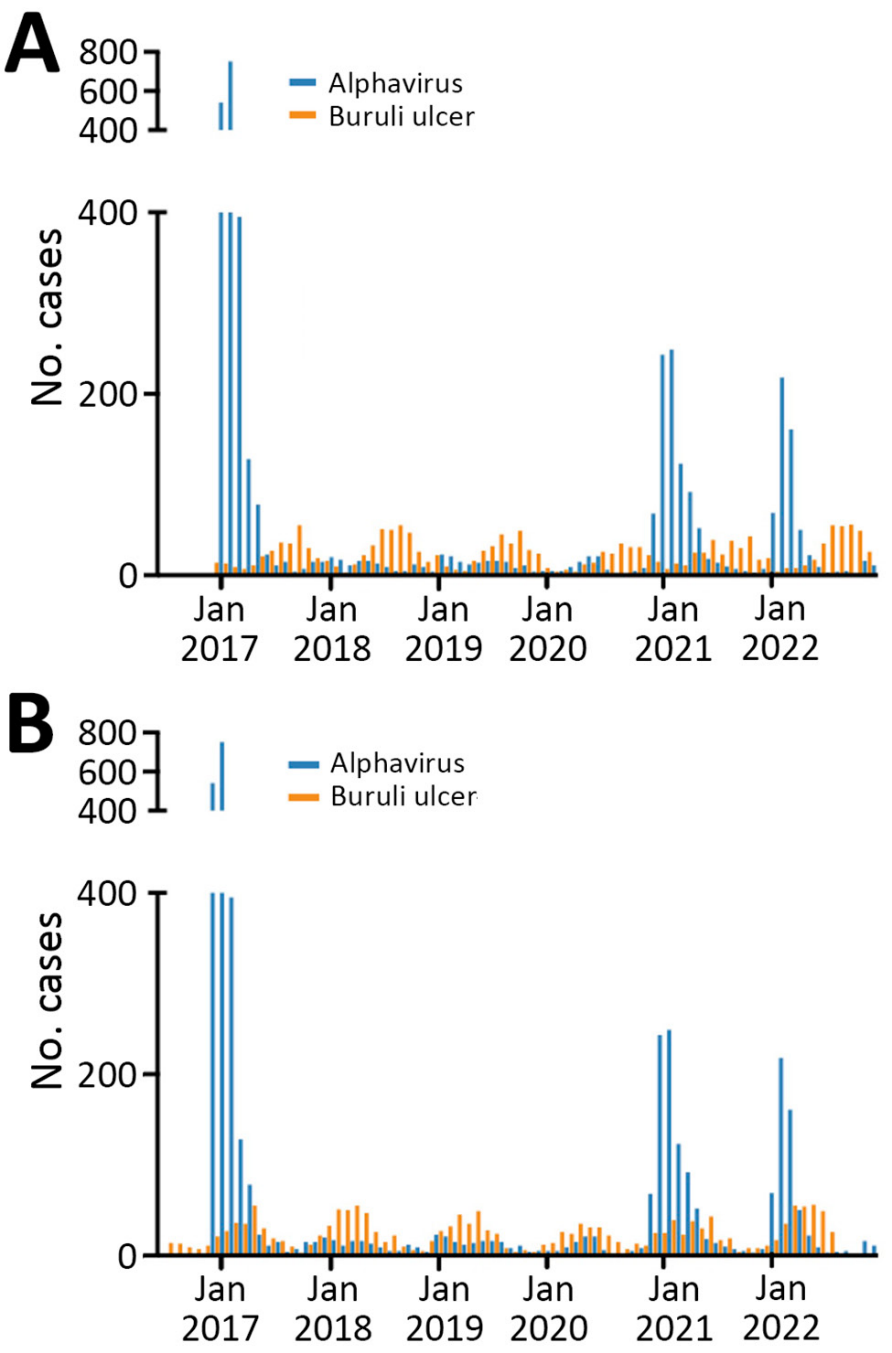
Alphavirus infection and Buruli ulcer notifications in Victoria, Australia, 2017–2022. A) Unadjusted month and year of notification. B) Month and year of notification manually adjusted for the known median Buruli ulcer incubation period of 5 months.

During 2017–2022, Victoria had 3,839 notified alphavirus infection cases. Notifications were strongly clustered by season (summer and autumn) and month. For Buruli ulcer, there were 1,761 notifications over the 6-year study period, also strongly clustered by month and season but peaking during winter and spring (Figure 1, panel A). The incubation period for Buruli ulcer has previously been calculated using interview information from case-patients who had only short exposures in Buruli ulcer–endemic areas. Two published estimates indicated medians of 4.5 and 5 months (*8*). To estimate and graphically illustrate the time offset between months of inferred transmission and notification, we assumed incubation period plus time to diagnosis/notification of 5 months for Buruli ulcer and 1 month for alphavirus infections (Figure 1, panel B). 

**Figure 2:**
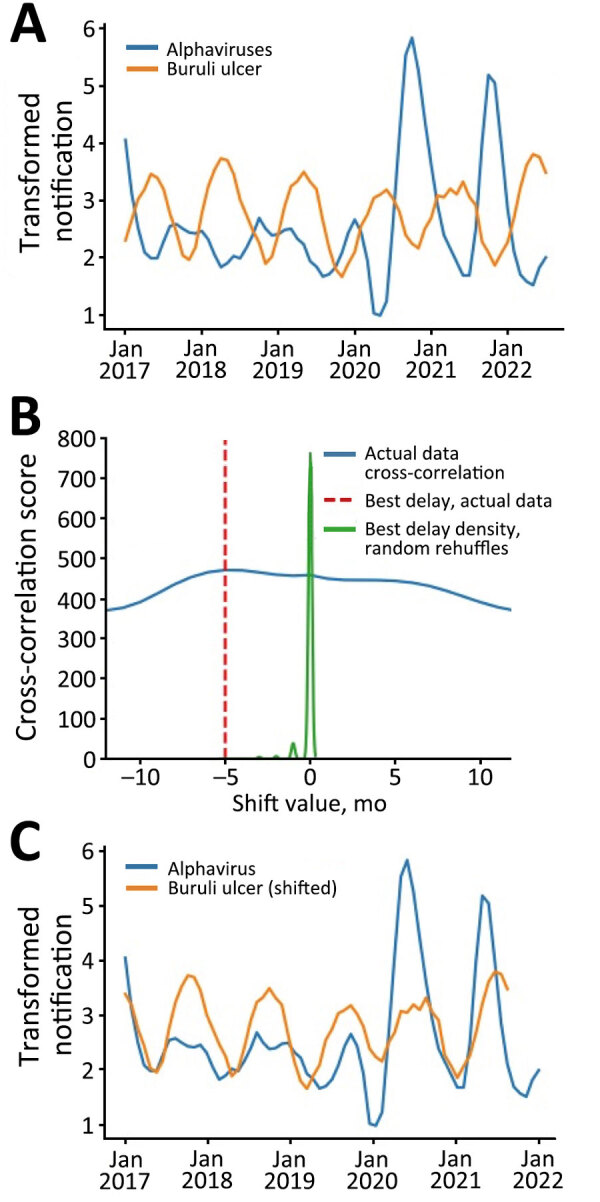
Analysis of temporally adjusted Buruli ulcer and alphavirus notifications in Victoria, Australia, 2017–2022. A) Notifications over time (no delay). Plot of the 2 datasets (outliers censored) was log transformed and smoothed by month and year. B) Optimal shift and cross-correlation analysis. Algorithmically determined cross correlation (blue line) and optimal curve shift of −5 months (vertical red dashed line) that best aligned the 2 log transformed curves shown in panel A. In green is a density curve that depicts the outcomes of 1,000 random data shuffles, serving as a visual indicator of how the observed −5 months curve shift diverges from random chance expectations. C) Notifications over time shifted to incorporate the Buruli ulcer incubation period of 5 months. Cross-correlation aligned smoothed log-transformed notification curves show synchronous inferred transmission peaks and troughs for both alphavirus infection and Buruli ulcer.

In addition, to test for confirmation bias, we reexamined the data using an observer-independent signal processing technique, cross-correlation, to analyze the relationship between the notification distributions. In the alphavirus time-series, the first 3 months of 2017 were identified as outliers using z-scores; therefore, we excluded those 3 timepoints from both datasets. The censored data series then underwent logarithmic transformation and smoothing to amplify the cyclic signal within the data. We displayed and analyzed data using Prism 10.0 (GraphPad, https://www.graphpad.com) or R (The R Project for Statistical Computing, https://www.r-project.org). The overlaid transformed series revealed an almost antiphase relationship between the Buruli ulcer and alphavirus signals, indicating the presence of similar but temporally offset cyclic patterns (Figure 2, panel A). 

We used the correlate function in the NumPy Python library (*9*) to identify the time-shift factor in months that optimized the correlation between the 2 signals. The algorithm identified –5 months as the optimal time shift. To test for statistical robustness, we performed the same cross-correlation analysis on 1,000 randomly reshuffled instances of the Buruli ulcer notification series. The resulting distribution from these iterations centered around 0, well separated from the optimal shift of –5 months (Figure 2, panel B). When we adjusted the Buruli ulcer notifications by that assumption-independent −5 months, we observed a close sinusoidal alignment with alphavirus notifications (Figure 2, panel C). 

Our results showed that inferred transmission of alphavirus infections and Buruli ulcer simultaneously reach their maximums during December–May (summer and autumn) and minimums during June–November (winter and spring) every year during the 6-year study period. The accepted explanation in temperate Victoria for variation by season in alphavirus infection notifications is that warmer weather provides necessary climatic conditions for increased prevalence of mosquito vectors. Even though animal reservoirs of alphaviruses are present throughout the year, transmission to humans falls to almost 0 during colder months. A recently published detailed quantitative observational study showed that *M. ulcerans* in possum excreta, and, by inference, in possums as wildlife reservoirs, is similarly present in the environment throughout the year (*10*). Hence, the previous paradigm of Buruli ulcer transmission by direct exposure to a stably contaminated environment does not fit well with the periodic notification patterns we observed. 

Our study addressed and offers evidence to resolve the question of correlation between alphavirus infection and Buruli ulcer notifications, but a wealth of other published research much more directly establishes a central role for mosquitoes in the transmission of Buruli ulcer in Victoria. That research includes evidence that human Buruli ulcer risk closely correlates with the proportion of mosquitoes PCR positive for *M. ulcerans* trapped in 7 towns on the Bellarine peninsula in Victoria during the mid-2000s (*11*). A survey of mosquitoes performed 15 years later on the Mornington peninsula in Victoria, on the opposite side of Port Phillip Bay from the original studies, demonstrated that 5.1/1,000 mosquitoes trapped there during 2016–2021 carried *M. ulcerans*. The more recent study also presented genomic evidence that *M. ulcerans* strains in mosquitoes were indistinguishable from the human and possum Buruli ulcer outbreak strains. In addition, the mosquito species most closely linked to Buruli ulcer transmission, *Aedes notoscriptus*, feeds on both humans and possums; some individual trapped mosquitoes simultaneously contained both human and possum blood (*12*). 

We have attempted to investigate alternative models of transmission that would explain the anatomic distribution of Buruli ulcer lesions we observed in Victoria (*13*), including variation in human skin temperature (*14*), and the hypothesis that outdoor exposure in Buruli ulcer–endemic areas leads to skin contamination with *M. ulcerans* (*15*). There was no support from those studies for an alternative model. 

## Conclusions

We conducted an analysis of statewide notifications of alphavirus infections and Buruli ulcer in Victoria, Australia, adjusted for incubation period. Our findings support other published evidence that Buruli ulcer is transmitted during the mosquito season by mosquitoes in this temperate region. 

Appendix Additional information on mosquitoes as vectors of *Mycobacterium ulcerans* based on analysis of notifications of alphavirus infection and Buruli ulcer, Victoria, Australia.
