# Supplementary material for: Mosquitoes as Vectors of Mycobacterium ulcerans Based on Analysis of Notifications of Alphavirus Infection and Buruli Ulcer, Victoria, Australia
Source: Emerg Infect Dis. 2024 Sep;30(9):1918–21. doi: 10.3201/eid3009.231073 (PMC11346997; doi:10.3201/eid3009.231073)
Supplement: Appendix — Additional information on mosquitoes as vectors of Mycobacterium ulcerans based on analysis of notifications of alphavirus infection and Buruli ulcer, Victoria, Australia. [file 23-1073-Techapp-s1.pdf]

Article DOI: https://doi.org/10.3201/eid3009.231073

EID cannot ensure accessibility for supplementary materials supplied by authors. Readers who have difficulty accessing supplementary content should contact the authors for assistance.

Mosquitoes as Vectors of *Mycobacterium ulcerans* Based on Analysis of Notifications of Alphavirus Infection and Buruli Ulcer, Victoria, Australia

Appendix

Appendix Table. Notifications (cases/month) of Buruli ulcer and alphavirus infections, Victoria, Australia, January 2017 to December 2022

Month	Buruli ulcer notifications	Alphavirus infection notifications
2017 Jan	14	542
2017 Feb	13	751
2017 Mar	9	395
2017 Apr	7	128
2017 May	11	78
2017 Jun	21	23
2017 Jul	27	11
2017 Aug	36	15
2017 Sep	35	4
2017 Oct	55	7
2017 Nov	30	15
2017 Dec	19	15
2018 Jan	16	20
2018 Feb	10	17
2018 Mar	3	11
2018 Apr	12	16
2018 May	22	16
2018 Jun	33	13
2018 Jul	51	9
2018 Aug	50	5
2018 Sep	55	5
2018 Oct	47	12
2018 Nov	26	9
2018 Dec	15	4
2019 Jan	22	23
2019 Feb	10	21
2019 Mar	6	15
2019 Apr	5	12
2019 May	16	14
2019 Jun	27	16
2019 Jul	32	16
2019 Aug	45	15
2019 Sep	35	8
2019 Oct	49	11
2019 Nov	28	4
2019 Dec	24	5
2020 Jan	8	5
2020 Feb	4	5
2020 Mar	6	9
2020 Apr	4	15
2020 May	12	21
2020 Jun	14	21
2020 Jul	26	6

Month	Buruli ulcer notifications	Alphavirus infection notifications
2020 Aug	24	2
2020 Sep	35	0
2020 Oct	31	5
2020 Nov	31	8
2020 Dec	22	68
2021 Jan	15	243
2021 Feb	7	249
2021 Mar	13	123
2021 Apr	11	92
2021 May	25	52
2021 Jun	25	18
2021 Jul	39	14
2021 Aug	23	10
2021 Sep	38	7
2021 Oct	30	5
2021 Nov	43	3
2021 Dec	17	7
2022 Jan	19	69
2022 Feb	4	218
2022 Mar	8	161
2022 Apr	8	50
2022 May	11	22
2022 Jun	17	9
2022 Jul	35	3
2022 Aug	55	4
2022 Sep	54	5
2022 Oct	56	2
2022 Nov	49	16
2022 Dec	26	11
